# A Systematic Review and Meta-Analysis of Combined Antibiotic Spacer with Ilizarov Methods in the Treatment of Infected Nonunion of Tibia

**DOI:** 10.1155/2021/6668617

**Published:** 2021-01-16

**Authors:** Zhibo Deng, Yuexi Mu, Xianding Sun, Yongqing Xu, Fubing Li, Liangjun Yin

**Affiliations:** ^1^Department of Orthopaedic Surgery, Second Affiliated Hospital of Chongqing Medical University, Chongqing, China; ^2^Department of Orthopaedic Surgery, 920th Hospital of Joint Logistics Support Force, Kunming, Yunnan Province, China

## Abstract

**Background:**

The objective of this systematic review was to evaluate current studies available reporting the antibiotic spacer combined with Ilizarov methods in the treatment of infected nonunion of tibia and to perform meta-analysis of bone results and infection recurrence to assess the efficacy of an antibiotic spacer combined with Ilizarov methods.

**Methods:**

The MEDLINE, Embase, Cochrane Library, CNKI, and CBM (Chinese Biological Medicine) databases were searched for articles published between January 2000 and July 2020. Assessment of study quality was performed using a modified version of the Newcastle-Ottawa scale. Effect size and 95% confidence intervals were calculated for the main outcome. Heterogeneity was assessed. Fixed-effect modeling and Stata version 15.1 were used to analyze the data. Sensitivity analyses were conducted with the evidence of heterogeneity.

**Results:**

11 studies involving 210 patients with infected nonunion of tibia were finally included in our meta-analysis. Bone results and infection recurrence were analyzed based on the single-arm meta-analysis. The average of external fixation index (EFI) was 46.88 days/cm in all studies included. The excellent rate in bone results and the rate of infection recurrence was 65% (95% CI: [0.22, 0.97], *I*^2^ = 0.0%, *P* = 0.932) and 6.99% (95% CI: [0.052, 0.325], *I*^2^ = 0.0%, *P* = 1.000) in patients with infected nonunion of tibia treated with an antibiotic spacer combined with Ilizarov methods.

**Conclusions:**

Our meta-analysis revealed that the patients with infected nonunion of tibia treated with an antibiotic spacer combined with Ilizarov methods had a high rate of excellent bone results and a low rate of infection recurrence. Therefore, combining the antibiotic spacer with Ilizarov methods may be an applicable choice for repairing and reconstructing infected nonunion of tibia.

## 1. Introduction

Infected nonunion of tibia is a common complication after open tibial fracture caused by high-energy trauma and is a difficult problem for orthopedic doctors all over the world [1]. It needs long hospital stay and a high cost, leading to burden on both patients and society, of which the final result is often amputation [2, 3]. Current recognized methods for cases with large defects are autologous bone graft with blood vessels, allograft, bone transport, and the Masquelet technique [4].

The vascular bone graft has the disadvantage of donor site damage and the risk of failure of the recipient site [5]. Because of the high cost of allograft transplantation, it will bring financial pressure to patients with large bone defects. For the Masquelet technique, also known as the induced membrane technique, although it has been used for various bone defects caused by infection, trauma, and tumors [6], a few studies have reported that the results are not satisfactory in severe complex open fractures [7, 8]. The Ilizarov technique can compensate for the bone defects left by excision of the infected bone with bone transport and reconstruct the length and structure of the limb. For patients with large segmental tibial defects, it is more effective, safer, and of low expense [9, 10]. Thus, the Ilizarov technique is now considered to be an ideal surgical treatment [11]. But the Ilizarov bone transport still has the possibility of infection recurrence [12]. Antibiotic cement can be used as a spacer for the bone loss area, forming soft tissue tunnel for bone transport and forming a self-induced membrane around the bone cement, which is conducive to bone regeneration [13].

Although the Ilizarov methods combined with the antibiotic spacer for the treatment of infected nonunion of tibia in clinical work have yielded satisfying results in most studies, in some studies, the opposite conclusion has been drawn [14]. Therefore, it is necessary to make a systematic summary on whether the antibiotic spacer combined with Ilizarov methods in the treatment of infected nonunion of tibia is worth recommending. However, no systematic research has been established. Therefore, we conducted a systematic review and meta-analysis of the relative literature to assess and quantitate the true effect and draw valuable conclusions.

## 2. Materials and Methods

### 2.1. Search Strategy

The MEDLINE, Embase, Cochrane Library, CNKI, and CBM (Chinese Biological Medicine) databases were searched for articles published between January 1, 2000, and July 30, 2020. The search strategy included following main search terms: “infected nonunion OR infection” AND “antibiotic cement OR PMMA OR spacer” AND “Ilizarov method OR Ilizarov technique OR bone transport” and so on. We also manually retrieved reference lists from the identified studies and relevant review studies for additional studies. Assessment of study quality was performed using a modified version of the Newcastle-Ottawa scale for included studies.

### 2.2. Selection Criteria

To minimize difference, studies were included if they met the following criteria:

1. *Article types*: original articles of randomised controlled trial (RCT), retrospective or prospective trials, and reported more than five cases
2. *Target population*: patients with infected defect or nonunion of the tibia with or nonsoft-tissue defects, age > 16 years old and <60 years old
3. *Intervention*: managements combined Ilizarov circular external fixator with antibiotic spacer, Ilizarov methods including bone transport, acute compression and lengthening, and compression osteosynthesis
4. *Outcomes*: the data of the eligible patient was complete

The exclusion criteria were as follows:

1. Article types were conference abstracts, letters, meta-analyses, case reports, or reviews
2. Full text was not available and studies with ambiguous results
3. Duplicates of previously published papers
4. Studies that included children (<16 years old) and the aged (>60 years old)

In addition, if the sources of the study population overlapped in two or more articles, only the study with the larger number of participants or the most recent study was included.

### 2.3. Data Extraction

We extracted the following data from the included articles.

1. *Basic information*: first author, publishing date, country, publishing journal, number of patients, demographic data of participants including age and gender, and mean previous surgical procedures, mean length of the bone defect, and mean length of follow-up
2. *Techniques*: design type and administration approach
3. *Outcomes*: bone results evaluated by the Paley method (rated as excellent, good, fair, and poor), functional results evaluated by the Paley method, complications per patient, external fixation time, and external fixation index reported in days/cm (EFI), infection recurrence, and other complications (pin-track infection, axial deviation, bone grafting, loosening of wires, breakage of wires, malunion, refracture, knee stiffness, ankle stiffness, amputation, limb edema, and peroneal nerve palsy et al.)

All the relevant data meeting the inclusion criteria were extracted independently by 2 authors, and any disagreement between them was resolved by discussion with each other.

### 2.4. Statistical Analysis

The pooled result of incidence rate of bone results, functional results, and complications in each included studies were analyzed by using the data-processing software Stata 15.1. Differences were expressed as the effect size (ES) with 95% confidence interval (CI) for the rate meta-analysis. Since in the most included studies, the incidence rate of the original data was not in the range of 30% to 70%, it was firstly converted by the arcsine transformation method to make it conform to the normal distribution. Then, the conclusion was restored using the formula *P* = (sin(*x*/2))^2^ to reach the final version. Statistical heterogeneity among studies was assessed using the standard chi-square test and *I*^2^ statistic. *I*^2^ > 50% was considered to have significant heterogeneity and the random-effect model was used, while *I*^2^ < 50% was considered to have low heterogeneity and the fixed-effect model can be used. Other major data extracted in this research were statistically analyzed using weighted means based on the sample size of each study by SPSS 23.0, including number of patients, mean age, mean previous surgical procedures, mean length of follow-up, mean length of the bone defect, external fixation time, external fixation index (EFI), and complications per patient. Sensitivity analysis was performed to determine if the results showed differences.

## 3. Results

### 3.1. Included Literature

**Figure 1:**
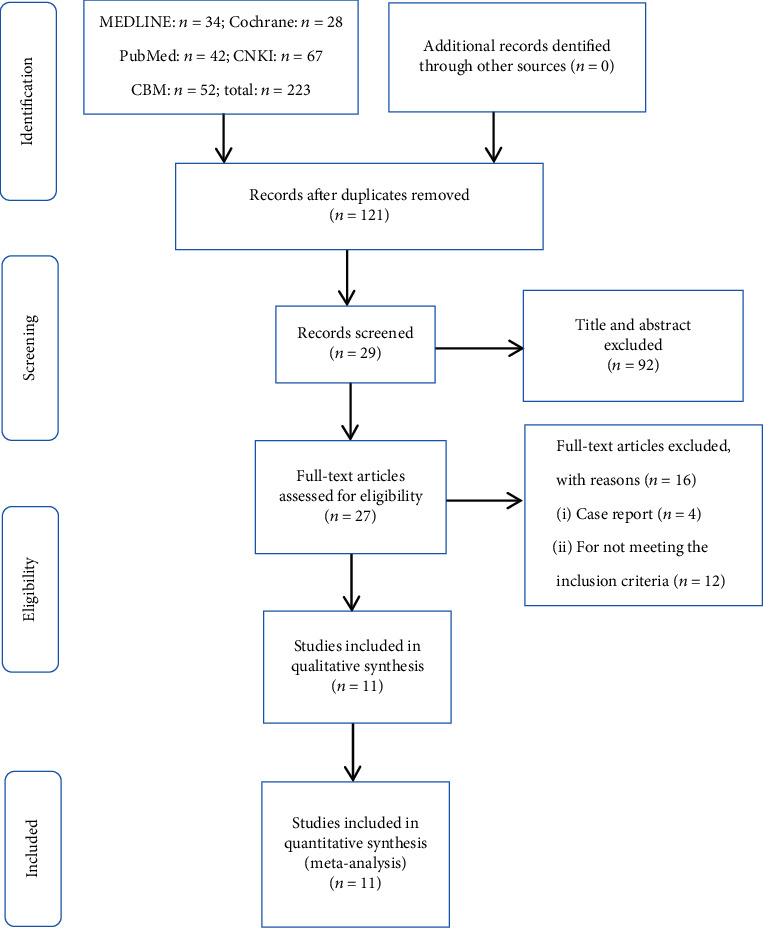
The flow chart of literature screening.

As shown in Figure 1, initially, a total of 223 potentially relevant articles were identified from the databases, in which 121 were screened. After screening the titles and abstracts, 118 were excluded. A total of 27 full-text articles were assessed for eligibility, while 16 were excluded for different reasons. Ultimately, 11 studies met the inclusion and exclusion criteria in our systematic review [14–24]. Of the included studies, 6 studies were retrospective case series [15, 18–20, 22, 23], 4 researches were retrospective comparative studies [14, 17, 21, 24], and 1 study was a prospective case series [16]. The quality of the included studies using a modified Newcastle-Ottawa scale (score range, 0-7) ranged from 5 to 7, with 5 positive answers taken to define a good quality study. The total scores were mainly 5 or 6, corresponding to moderate quality. Overall, the quality of the eleven included studies was moderate. The detailed assessment is shown in Table 1.

### 3.2. Patient Information

The studies were published between 2004 and 2019. A total of 210 patients with infected nonunion of tibia treated by combined antibiotic spacer with Ilizarov methods were included in our study. The mean age of all patients was 36.5 years. Patients had an average of 5.46 previous surgical procedures before receiving the treatment of Ilizarov methods combined with antibiotic spacer [14, 19, 20]. The mean length of the bone defect in the patients was 7.92 cm [14, 16–23]. The mean length of follow-up was 27.4 months in the patients [14–16, 18–20, 22, 23].  Table 2 summarizes the baseline characteristics of the included studies and patients.

### 3.3. Interventions and Outcomes

The intervention consisted of three main steps: radical surgical debridement, implantation of antibiotic-impregnated spacer, and Ilizarov methods. The Ilizarov methods included three techniques: bone transport, acute shortening and relengthening, and compression osteosynthesis. Except for one study [19] where the spacer was made of antibiotics and calcium sulfate, the spacers in the remaining studies were all made of antibiotics and bone cement. The shape of the spacer can be columnar or bead-like. Bone grafting as a routine treatment was recommended at the end of the bone transport in 2 included studies [16, 17]. Suturing the induced membrane produced by the antibiotic spacer was reported in 3 included studies [18, 20, 21].

In the outcomes of included studies for infected nonunion of tibia, the average of the bone union rate was 94.67%. The mean complications of every patient were 0.86. The mean external fixation time was 13.18 months [15, 16, 18–21]. The mean external fixation index was 46.88 days/cm [16, 18–21]; further details are listed in Table 3. Details of major related complications are shown in Table 4.

### 3.4. Bone Results

The criteria recommended by Paley were adopted to evaluate clinical bone results in the studies [14, 17, 19, 20, 22]. Bone results were evaluated by 4 criteria: union, infection, deformity, and limb-length discrepancy.

1. *Excellent*: fracture healing and three of the following indicators—no recurrent infection, local deformity less than 7°, and unequal length of limbs less than 2.5 cm
2. *Good*: fracture healing and any two of the 3 indicators above
3. *Fair*: fracture healing and any one of the 3 indicators above
4. *Poor*: fracture did not heal or fracture again or no match for any of the 3 indicators above [9]

**Figure 2:**
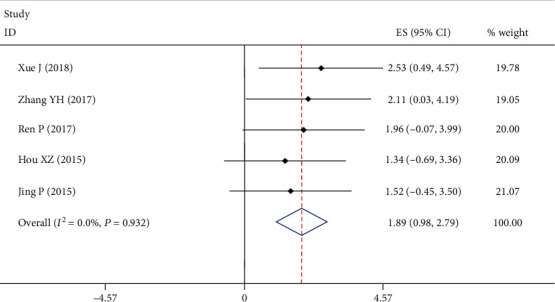
Forest plot showing excellent rate of bone results. According to the formula (*P* = (sin(*x*/2))^2^), the final rate was equal to 0.65; 95% CI ranged from 0.22 to 0.97.

5 studies with 107 patients reported the excellent rate in bone results. Since the heterogeneity test indicated no significant heterogeneity (*I*^2^ = 0.0%), a fixed-effect model was used. The meta-analysis showed that the excellent rate in bone results was 65% (95% CI: [0.22, 0.97], Figure 2).

### 3.5. Infection Recurrence

**Figure 3:**
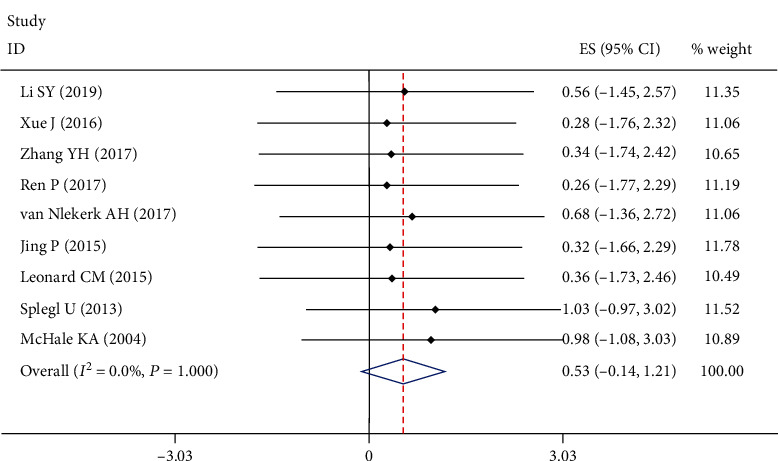
Forest plot showing rate of infection recurrence. According to the formula (*P* = (sin(*x*/2))^2^), the final rate was equal to 0.0699, 95% CI ranged from 0.0518 to 0.325.

10 studies with 164 patients reported the infection recurrence in complications. Since the heterogeneity test indicated no significant heterogeneity (*I*^2^ = 0.0%), a fixed-effect model was used. The meta-analysis showed that the rate of the infection recurrence was 6.99% (95% CI: [0.052, 0.325], Figure 3) after the antibiotic spacer combined with Ilizarov methods was applied to the infected nonunion of tibia.

### 3.6. Sensitivity Analysis

Although the heterogeneity test indicated that no significant heterogeneity was found (*I*^2^ = 0.0%), our research mainly includes retrospective case studies and cohort studies. To test the stability of the results of our study, we performed the sensitivity analysis. The results of the sensitivity analysis showed minor changes in ES and 95% CI when one study was randomly removed from the meta-analysis, demonstrating that the results of our research are less sensitive and have good stability.

## 4. Discussion

As far as we acknowledge, this is the first systematic review about infected nonunion of tibia treated by an antibiotic spacer combined with Ilizarov methods. This systematic review included 11 studies, and we conducted a meta-analysis of the 11 studies to evaluate the actual efficacy of the antibiotic spacer combined with Ilizarov methods in the treatment of infected nonunion of tibia. The excellent rate in bone results was 65% (95% CI: [0.22, 0.97]; *I*^2^ = 0.0%, *P* = 0.932). The reinfection rate after surgery was 6.99% (95% CI: [0.052, 0.325], *I*^2^ = 0.0%, *P* = 1.000). And the data were not statistically heterogeneous. Therefore, our results showed that the patients with infected nonunion of tibia treated by antibiotic spacer combined with Ilizarov methods had a high rate of excellent bone results and a low rate of infectious recurrence.

According to a tibial anatomical study [25], besides less local soft tissue cover, the lower 2/3 of the tibial luminal diaphysis has no vascular foramen, and only a small hole exists in the posterior side of the junction of the upper 1/3 of the tibial luminal diaphysis. If a fracture occurs at the junction of the middle and lower 1/3 of the tibia, it can damage the nutrient artery, resulting in reduced blood supply to the lower 1/3 of the tibia. This predisposes the breeding of a large number of bacteria that is difficult to be cleared by the body, which can even form bacterial emboli leading to vascular blockage at the fracture end, tibial ischemia and necrosis, and local infection and purulence, evolving into chronic osteomyelitis eventually. Although infected nonunion of the tibia needs radical debridement to remove infected and necrotic bone, bacteria may still hide in the bone and soft tissue lacunae and cause repeated infection during the distraction process [9, 26]. Therefore, control of bacterial infection is also an important part in the treatment of infected nonunion of tibia. Currently, the main method of infection control was to select sensitive antibiotics according to the results of drug sensitivity and to take systemic administration [27]. However, the dosage of this method was large, and the adverse drug reactions are obvious. Furthermore, it is easy to cause drug resistance. More importantly, tibias with chronic osteomyelitis have poor local blood supply. It is difficult for systemic antibiotics to reach an effective concentration at the lesion site, so the purpose of infection control cannot be achieved [2].

After the local application of an antibiotic spacer, the local concentration of antibiotics was nearly 200 times higher than that of systemic administration. It is enough to kill drug-resistant bacteria. Moreover, bone cement can provide local support and facilitate early functional exercise [28], although there is no standard for the use of antibiotics in bone cement, because excessive addition will reduce the mechanical strength. But a study found that adding 8 grams of antibiotics to 40 grams of bone cement is still safe [29].

Radical debridement and the antibiotic spacer can effectively eliminate the infection, but the bacteria residue and subsequent bone nonunion are other major problems for orthopedic surgeons [30]. At present, there are many reports recording the treatment of tibial defects with Ilizarov methods. Ilizarov methods based on the biological principles of distraction osteogenesis, solving the problems of bone defects and tissue loss in patients [31], have gradually become the main method for the treatment of infected nonunion of tibia.

The popularity of the Ilizarov technique is mainly due to the improvement of local blood supply, which can not only promote fracture healing but also enhance the ability to control infection. Meanwhile, for patients with a local defect of soft tissue in the injured area, bone transport can promote soft tissue to repair, which is also better than the Masquelet technology in this respect [32], since the bone is distracted to an appropriate length every day, the surrounding soft tissue regenerates along with the pulling force during bone transport, and the stimulation during bone transport for blood vessel and peripheral nerve around the defective area is also a contributing factor to the repair of soft tissue.

Therefore, the systematic review was conducted to estimate whether combined antibiotic cement spacer with Ilizarov methods in the treatment of infected nonunion of the tibia is an ideal approach. According to our meta-analysis, the rate of infection recurrence is lower than the rate in the study by Yin et al. using other treatments [11]. Besides, the rate of infection recurrence is also lower than 9.3% in the meta-analysis of the Masquelet technique by Morelli and colleagues [33].

Considering the external fixation index in our series (mean 46.88 days/cm), as well as the 49.2 days/cm reported in the meta-analysis of Ilizarov methods by Yin et al. [11], it appears that the application of antibiotic spacers does not significantly increase the period of external fixation. Meanwhile, the excellent rate in bone results was 65%. Compared with 61% in Yin et al. [11] only using Ilizarov methods, the treatment effect and satisfaction were improved.

Although this study comprehensively and systematically evaluated the efficacy of the antibiotic spacer combined with Ilizarov methods, there are still some limitations in our study. (1) The sample size in the studies involved was small, and most studies were retrospective studies. (2) The included researches lacked standardised and unified protocols for the process of treatment, especially the removal timing and removal method of the spacer. (3) Due to the lack of records of bone and functional results, many variables of included studies cannot be analyzed in combination, all of which may lead to bias in conclusions. Thus, further research based on prospective, large-size, and multicentre clinical study should be done.

Of course, some scholars believe that Ilizarov methods also have some shortcomings [34]: (1) The external fixator is clumsy, and wearing it for a long time will affect the patient's daily activities. (2) Due to the long time of treatment and slow speed of mineralization, a longer time is often needed to enhance the mechanical strength of the new bone segment. (3) The needle passage of the external fixator can provide an infection channel for bacteria. In order to avoid the occurrence of needle passage infection, frequent daily care for the needle passage is needed. (4) After bone transport, the docking site may not heal well, and it may even require surgery again. (5) In the process of bone transport, persistent pain can be caused by increased pressure in the soft tissue, continuous nerve stimulation and the inevitable occurrence of skin cutting. Therefore, it is necessary to comprehensively evaluate various factors, to select appropriate indications, and to flexibly apply different methods in order to complement each other's advantages and achieve the best effect.

## 5. Conclusions

Our research revealed that patients with infected nonunion of tibia treated by antibiotic spacer combined with the Ilizarov methods had a high rate of excellent bone results and low rate of infection recurrence. Therefore, combined antibiotic spacer with Ilizarov methods may be an applicable choice for repairing and reconstructing infected nonunion of tibia.

## Figures and Tables

**Table 1:** Evaluation of included studies using modified Newcastle-Ottawa scale.

Parameter	Study										
	Wang H (2019)	Li SY (2019)	Xue J (2018)	Zhang YH (2017)	Ren P (2017)	van Niekerk AH (2017)	Hou XZ (2015)	Jing P (2015)	Leonard CM (2015)	Spiegl U (2013)	McHale KA (2004)
Study population clearly defined	Yes	Yes	Yes	Yes	Yes	Yes	Yes	Yes	Yes	Yes	Yes
Consecutive patients included	Yes	Yes	Yes	Yes	No	Yes	Yes	Yes	Yes	Yes	Yes
Patients represent population with infected nonunion of tibia	Yes	Yes	Yes	Yes	Yes	Yes	Yes	Yes	Yes	Yes	Yes
Accurate description of infected nonunion of tibia	Yes	Yes	Yes	No	Yes	Yes	Yes	Yes	Yes	Yes	Yes
Assessment of outcome well defined	Yes	No	Yes	Yes	Yes	No	Yes	Yes	No	No	No
Complications well defined	No	Yes	Yes	Yes	Yes	Yes	No	Yes	Yes	Yes	Yes
Follow-up long enough for outcomes to occur (>1.5 years)	No	Yes	No	Yes	Yes	No	No	Yes	Yes	Yes	Yes

Studies with 5 positive answers were defined as good quality.

**Table 2:** Characteristics of included studies.

Study no.	First author	Year	Study design	Number of patients	Men/women	Age (years)	Mean previous operations (per patient)	Mean bone defects (cm)	Mean time from initial treatment (months)	Follow-up (months)
1	Wang [24]	2019	RC	31	18/13	44.68	7.5	—	7.48	—
2	Li [23]	2019	RS	18	11/7	41.2	18	11.8	11.1	21.5
3	Xue [22]	2018	RS	12	9/3	31.5	—	10	—	14.5
4	Zhang [14]	2017	RC	8	—	41.3	3.1	6.4	22.8	31
5	Ren [20]	2017	RS	14	12/2	40.4	3.3	8.5	15.3	17.5
6	van Niekerk [21]	2017	RC	12	—	35.1	—	5.0	—	—
7	Hou [17]	2015	RC	15	15/0	31.28	—	4	10.29	—
8	Jing [19]	2015	RS	58	38/20	29.4	6.3	9.5	30.5	31.6
9	Leonard [18]	2015	RS	7	—	29	—	7	3	28
10	Spiegl [16]	2013	PS	25	22/3	46	—	5.3	9.6	29.4
11	McHale [15]	2004	RS	10	8/2	31	—	—	—	36

Abbreviations: RS: retrospective case series; RC: retrospective comparative study; PS: prospective case series.

**Table 3:** Interventions and outcomes of included studies.

Study no.	The process of spacer	Bone results (Paley) (excellent/good/fair/poor)	Functional results (Paley) (excellent/good/fair/poor)	EFT (months)	EFI (cm/d)	Complications (per patient)	Ingredients of spacer
1	After RD, antibiotic cement beads were implanted. 2 weeks later, spacer is gradually removed during bone transport.	—	22/7/2/0	1.7	—	—	Cement mixed with sensitive antibiotics
2	After RD, antibiotic cement was implanted. After 6 weeks, the spacer was removed before bone transport.	—	—	8.5	—	0.61 (11/18)	20 g cement mixed with 3-4 g vancomycin
3	After RD, antibiotic spacers containing calcium sulfate were implanted and eventually absorbed naturally.	11/1/0/0	—	6	—	0.44 (12/27)	Solid containing calcium sulfate powder and vancomycin
4	After RD, antibiotic cement beads were implanted. Spacer is gradually removed during bone transport.	7/1/0/0	—	4.4	—	2.25 (18/8)	20 g cement mixed with 0.25 g gentamicin and 3 g vancomycin
5	After RD, antibiotic bone cement was implanted. After 4-7 weeks, the antibiotic cement was removed and induction membrane was sutured. The bone transport will begin after 7 days.	—	—	13.2	48.5	1.43 (20/14)	10 g cement mixed with 1 g vancomycin
6	After RD, antibiotic bone cement was implanted. After 6 weeks, the antibiotic cement was removed and induced membrane was sutured. Besides, the bone transport began.	—	—	8.5	56.6	0.5 (6/12)	Antibiotic-impregnated cement
7	After RD and bone grafting, the remaining defects were filled with bone cement containing gentamicin. After 1 week, the spacer was removed before bone transport.	6/8/1/0	—	7.5	—	—	Cement mixed with gentamicin
8	After RD, antibiotic bone cement was implanted. After 7-10 days, spacer was gradually removed during bone transport.	30/23/5/0	28/18/12/0	10.6	36	0.67 (39/58)	10 g cement mixed with 1.2 g tobramycin and 0.5 g vancomycin
9	After RD, antibiotic bone cement was implanted. After 9 weeks, the spacer was removed and induced membrane was sutured. Besides, the bone transport began.	—	—	18	81	1.43 (10/7)	40 g cement mixed with 0.5 g gentamicin or 2 g vancomycin
10	After RD, implant antibiotic cement. The spacer was removed, and the Ilizarov method was used until no bacteria were found in all bacterial cultures of tissue samples. The spacer was removed, and the Ilizarov methods were performed.	—	—	21.7	57	1.4 (35/25)	Antibiotic-impregnated cement
11	After RD, antibiotic cement beads were implanted. After 3 weeks, the spacer was removed before performing Ilizarov methods.	—	—	9	—	0.3 (3/10)	2 g gentamicin in a bag of bone cement

Abbreviations: RD: radical debridement; EFT: external fixation index; EFI: external fixation index.

**Table 4:** List of related complications.

Complications	Number of patients
Pin tract infection or pin loosening	53
Axial deviation	21
Delayed union	6
Bone grafting	4
Joint stiffness	10
Malunion	12
Amputation	2
Refracture	4
Muscle contractures	4

## Data Availability

All data used to support the findings of this study are included within the article.

## Abbreviations

95% CI: 95% confidence interval
CNKI: China National Knowledge Infrastructure
EFI: External fixation index.
